# Regenerative Endodontic Treatment in Permanent Incisors: Two Case Reports with 6 Years of Follow-Up

**DOI:** 10.3390/children13020246

**Published:** 2026-02-10

**Authors:** María Biedma-Perea, Marcela Arenas-González, María José Barra-Soto, Carolina Caleza-Jiménez, David Ribas-Pérez

**Affiliations:** Department of Stomatology, Paediatric Dentistry, Faculty of Dentistry, University of Seville, C/Avicena SN, 41009 Seville, Spain; mbiedma1@us.es (M.B.-P.); mbarra@us.es (M.J.B.-S.); ccaleza@us.es (C.C.-J.); dribas@us.es (D.R.-P.)

**Keywords:** apexification, case report, immature teeth, regenerative endodontic

## Abstract

**Highlights:**

**What are the main findings?**

- Regenerative endodontic treatment achieved long-term periapical healing and structural reinforcement in three immature permanent incisors;
- Both triple antibiotic paste and calcium hydroxide protocols resulted in stable calcified apical barriers over more than six years of follow-up.

**What are the implications of the main findings?**

- Regenerative procedures represent a reliable alternative for managing necrotic immature teeth, even in cases where apexification has previously failed;
- Extended follow-up confirms that regenerative approaches can provide durable clinical stability and sustained root maturation in pediatric patients.

**Abstract:**

Background: Regenerative endodontic treatment (RET) has emerged as a biologically based alternative to traditional apexification for managing immature permanent teeth with pulp necrosis. By promoting tissue ingrowth and continued root development, RET aims not only to eliminate infection but also to reinforce structurally compromised roots. Although its clinical use has expanded, evidence regarding the long-term predictability and durability of RET remains limited, as most published studies provide only short- or mid-term follow-up. Case presentation: This report describes two pediatric cases involving regenerative procedures performed on three immature permanent maxillary incisors, each followed for more than six years. The first case involved a 7-year-old girl who developed pulp necrosis in a maxillary lateral incisor after acute dental trauma. Management followed a regenerative protocol using triple antibiotic paste (ciprofloxacin, metronidazole, and minocycline) as intracanal medication and mineral trioxide aggregate as the coronal barrier. The second case concerned an 8-year-old girl presenting with chronic infection and sinus tracts affecting both maxillary central incisors. These teeth were treated using a regenerative approach with calcium hydroxide as the intracanal medicament and Biodentine as the sealing material. Clinical, radiographic, and cone beam computed tomography evaluations demonstrated complete symptom resolution and periapical healing but incomplete progressive apical closure. All treated teeth developed a calcified apical barrier, and outcomes remained stable throughout the extended follow-up period. Conclusions: While inherently limited by the nature of case reports, these findings support RET as a reliable and durable therapeutic option for necrotic immature permanent teeth, including cases in which conventional apexification has not been successful.

## 1. Introduction

Interest in regenerative endodontic treatment (RET) has grown considerably since the first landmark cases reported by Iwaya et al. [1] in 2001 and Banchs and Trope [2] in 2004. In its early stages, this procedure was referred to as revascularization. Over time, several terms, such as regenerative endodontic treatment, revascularization, revitalization, and maturogenesis, have been used to describe the same biological approach [3,4,5]. The American Association of Endodontists (AAE) later adopted the term regenerative endodontic treatment [6], while the European Society of Endodontology (ESE) prefers to use revitalization in its official guidelines [3,4].

Regenerative endodontic treatment represents a true paradigm shift in managing immature teeth affected by pulp necrosis and/or apical periodontitis or abscess [7,8]. Through this technique, researchers have sought to activate the body’s inherent biological mechanisms to complete root development, including apical closure, giving rise to what are now known as regenerative endodontic procedures (REPs). These procedures aim to replace damaged or missing structures by promoting the formation of new, healthy tissues, ultimately restoring both the form and function of the pulp–dentin complex [9].

During the past decade, regenerative endodontic procedures have emerged as a practical and less invasive alternative, enabling complete root maturation in immature teeth. The core concept underlying these procedures is that a disinfected root canal, coupled with the induction of new vascular supply, can reestablish blood flow and support continued apical development [10,11]. As understanding of the biological mechanisms involved has further progressed, the gap between theoretical knowledge and clinical practice has progressively narrowed, positioning regeneration as one of the most promising and forward-looking fields in modern endodontics.

Effective control of microbial proliferation within the root canal system and the resolution of periapical or interradicular inflammation are essential for the success of RET. To achieve this, intracanal medication is commonly applied between clinical sessions to ensure a favorable prognosis in teeth with endodontic involvement [12]. However, there is still no consensus regarding the ideal medicament for this type of treatment [13].

Calcium hydroxide, Ca(OH)_2_, in an aqueous paste form has been widely used as an intracanal medicament due to its biocompatibility and its ability to stimulate periapical repair [14]. Nevertheless, its limited antimicrobial activity and low solubility restrict the diffusion of hydroxyl ions into deeper and infected areas, such as the periapical region [15]. To overcome these limitations, non-aqueous solvents such as polyethylene glycol (PEG-400) have been evaluated, as they prolong the release of Ca(OH)_2_ and reduce the need for multiple medication changes, showing favorable clinical outcomes [16,17,18].

Due to its highly alkaline pH, calcium hydroxide creates a hostile environment for microorganisms by altering their cellular membranes and denaturing proteins, demonstrating antibacterial efficacy against a wide range of species. However, its inability to completely eliminate resistant microorganisms, such as *Enterococcus faecalis* and *Candida albicans*, limits its effectiveness, particularly in anatomically complex areas such as deep dentinal tubules or lateral canals [19,20]. Its low solubility also restricts its diffusion and, consequently, its performance in endodontic retreatment cases.

Given the polymicrobial nature of endodontic infections and the difficulty of achieving complete eradication, several studies have explored the use of intracanal antibiotics to enhance antimicrobial activity [21,22]. Among the most widely used formulations is the triple antibiotic paste, composed of metronidazole, ciprofloxacin, and minocycline, which has demonstrated high efficacy in eliminating bacteria from infected dentin, necrotic pulps, and apical periodontitis [23]. However, its clinical adoption has been limited, mainly due to tooth discoloration caused by minocycline, prompting the search for alternative antibiotic agents [21,22,23,24].

Combined physical and chemical strategies are more effective than single interventions for eradicating *E. faecalis* and *C. albicans* in endodontic therapy. For *E. faecalis*, recommended approaches include intracanal medicaments (e.g., amoxicillin and clavulanate, double/triple antibiotic pastes), adjunctive photodynamic therapy and laser activation (Er:YAG, Er, Cr:YSGG), and novel agents such as graphene oxide or nanosilica antibiotic combinations [25]; for *C. albicans*, effective measures emphasize ultrasonic activation with antimicrobial gels, photodynamic therapy plus mechanical instrumentation, antifungal antiseptics (Octenisept, alexidine), and emerging modalities like cold atmospheric plasma and natural compounds (alpha-mangostin, nano-curcumin) [26]. Clinical choice should be tailored to canal anatomy, biofilm complexity, and patient factors, prioritizing multimodal protocols that improve irrigant penetration and biofilm disruption to optimize long-term outcomes.

One of the most promising alternatives is fosfomycin, an inhibitor of the enzyme MurA involved in bacterial cell wall biosynthesis [27]. This broad-spectrum antibiotic has shown synergistic effects when combined with other antimicrobial agents, allowing for dose reduction and minimizing toxicity [28,29,30,31]. Despite these encouraging findings, further studies are needed to determine the optimal concentrations required to maintain antimicrobial efficacy without causing cytotoxicity, as well as to develop delivery systems capable of prolonging therapeutic action [24]. Recently, natural compounds with antimicrobial and regenerative properties have been proposed as potential intracanal medicaments. Epigallocatechin gallate (EGCG), a polyphenol derived from green tea, has gained recognition for its wide therapeutic spectrum, including antimicrobial, anti-inflammatory, and antioxidant activities [32]. Although preliminary studies have shown promising results when used as an intracanal medicament, further research is required to evaluate its synergistic interactions with antibiotics and the performance of different delivery vehicles.

During the mechanical instrumentation of the root canal system, a smear layer approximately 1–2 µm thick is formed, which may become contaminated [33,34]. This layer can limit irrigants [35], which is why the use of chelating agents, such as ethylenediaminetetraacetic acid (EDTA), is recommended to remove the inorganic components of the smear layer [36].

As described in numerous studies, one of the main limitations in the field is the lack of adequate long-term follow-up of regenerative treatments, which restricts the ability to fully assess their durability, stability, and true clinical effectiveness over time. It is possible to find studies reporting outcomes at 3, 6, 9, and 12 months [37,38,39,40,41,42,43]; 18 months [44,45,46,47]; 24 months [48]; 28 months [49]; and just one with a 4-year follow-up [50]. For this reason, this work aims to present two clinical cases involving the regenerative treatment of three immature permanent teeth, with an extended follow-up period of over six years, in order to provide clinically relevant long-term data on treatment outcomes.

## 2. Case Report

### 2.1. Clinical Presentation

This study reports on a side-by-side comparison of two distinct clinical presentations of regenerative endodontic therapy. Differences center on the variability in procedural protocols and the resulting outcomes, interpreting these differences in light of current guidelines and the recent scientific literature. All procedures were carried out in full compliance with the ethical principles outlined in the Declaration of Helsinki (1964) and its subsequent amendments. Written informed consent was obtained from all participants prior to their inclusion in this study.

### 2.2. Clinical Case 1

**Figure 1:**
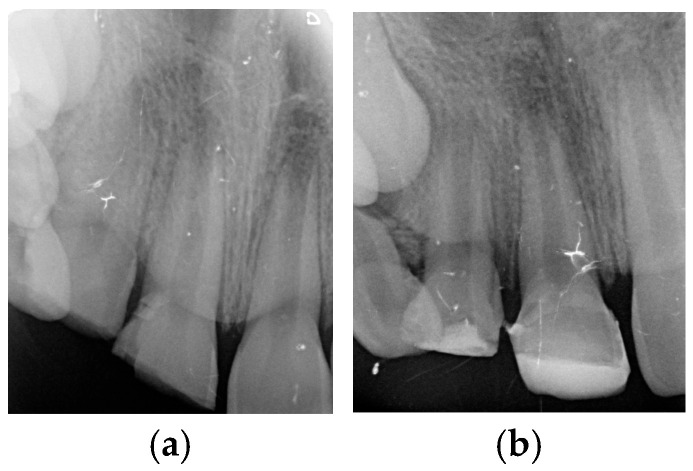
(**a**) Periapical radiograph taken during the initial visit. (**b**) The access cavity was provisionally sealed with composite material at the first appointment.

A 7-year-old girl attended the dental clinic after experiencing trauma more than 48 h earlier following a fall from a slide. During this time, no specific care was provided due to a lack of information. Extraoral clinical examination revealed no injury to the perioral or labial tissues. Intraoral examination showed enamel and dentin fracture in the maxillary right central incisor and enamel, dentin, and pulp fracture in the maxillary right lateral incisor. Periapical radiographic examination revealed an open apex measuring 3.7 mm (Nolla’s stage 8) [51] and thin dentinal walls (Figure 1a). Based on the clinical and radiographic findings, a regenerative procedure was planned for the maxillary right lateral incisor, as shown in Table 1.

At the first treatment appointment, an access cavity was prepared, allowing drainage of hemorrhagic exudate and confirming pulp necrosis. The root canal was irrigated slowly with at least 20 mL of 2.5% sodium hypochlorite (NaOCl), maintaining the needle (Miraject-Endotec Luer 23G 0.6 × 25 mm Hager & Werken GmbH &Co. KG, Duisburg, Germany) 1 mm short of the apex. The canal was then dried with sterile paper points. A paste composed of ciprofloxacin, metronidazole, and minocycline was prepared following the protocol described by Hoshino et al. [23], mixed to a creamy consistency, and placed into the canal using a Lentulo spiral. The access cavity was sealed temporarily with a resin composite (Figure 1b).

**Figure 2:**
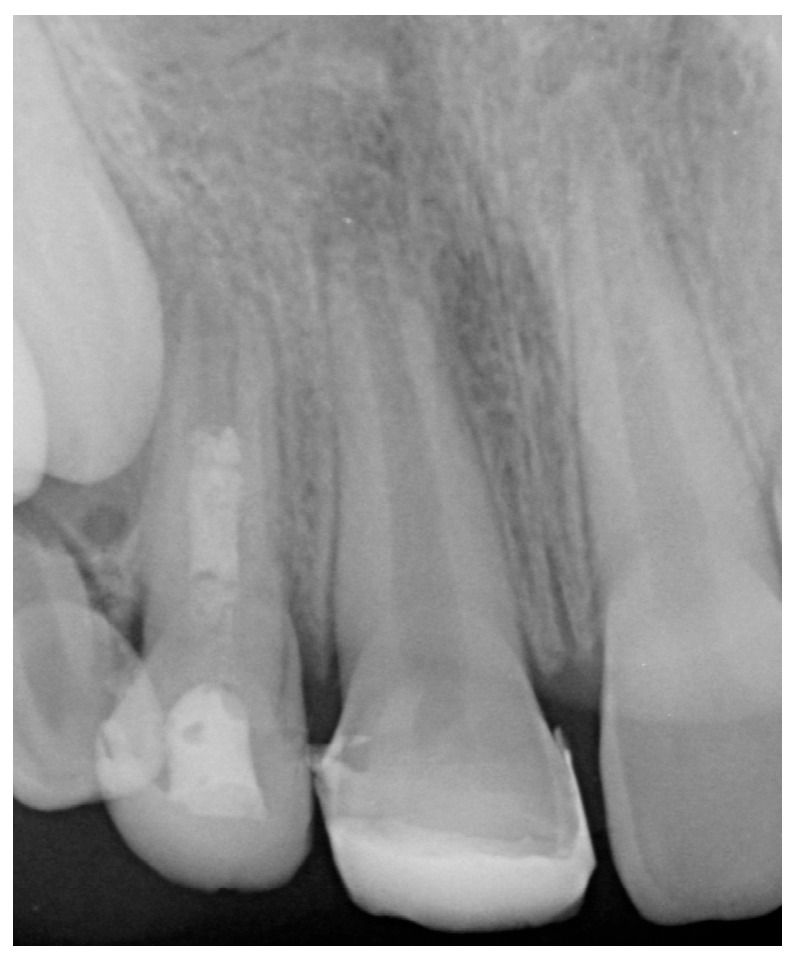
Clinical Case 1: Hermetic sealing with composite material at the second appointment.

At the second appointment, four weeks later, the access cavity was reopened. A sterile file was gently passed slightly beyond the working length to mechanically irritate the periapical tissues and induce bleeding into the canal. The bleeding was allowed to rise up to 3 mm below the CEJ and left for approximately 15 min to allow coagulum formation. After clot stabilization, a layer of mineral trioxide aggregate (MTA; Dentsply Tulsa Dental, Tulsa, OK, USA) was carefully placed over the blood clot, followed by a cotton pellet and coronal sealing with a resin composite (Figure 2).

**Figure 3:**
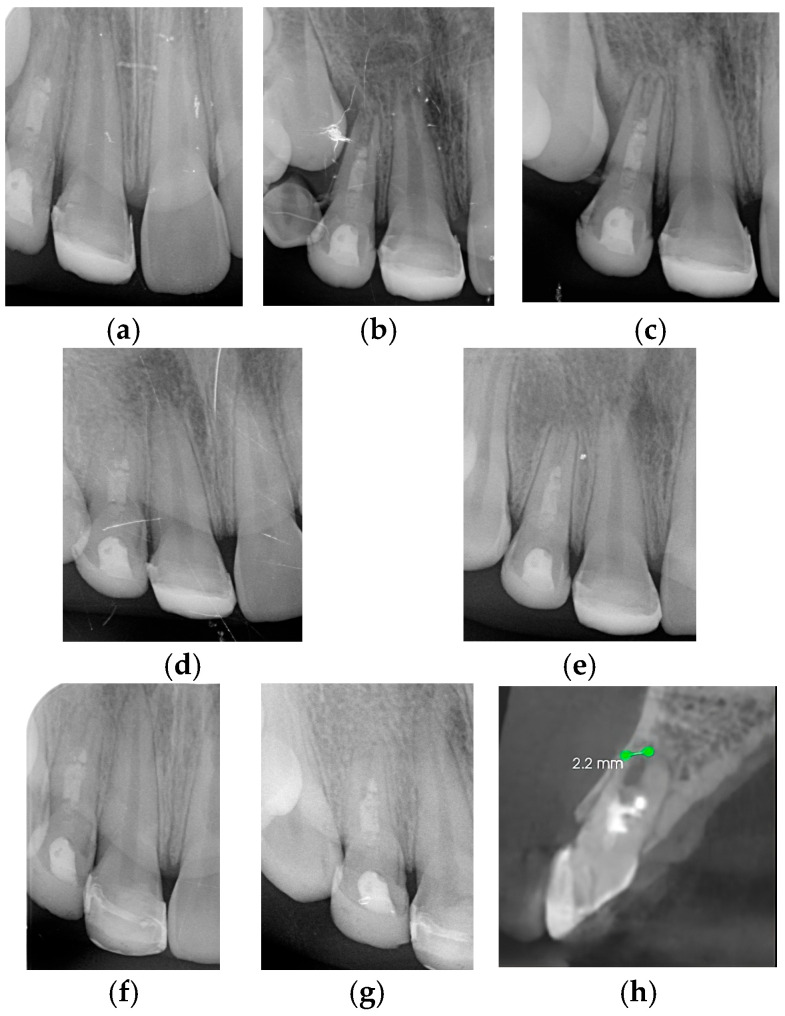
Clinical Case 1. (**a**) Periapical radiograph taken at 6-month follow-up. (**b**) Periapical radiograph taken at 1-year follow-up. (**c**) Periapical radiograph taken at 2-year follow-up. (**d**) Periapical radiograph taken at 3-year follow-up. (**e**) Periapical radiograph taken at 4-year follow-up. (**f**) Periapical radiograph taken at 5-year follow-up. (**g**) Periapical radiograph taken at 6-year follow-up. (**h**) CBCT taken at 6-year follow-up.

At the third appointment, esthetic restoration of the maxillary right lateral incisor was performed to recover its original shape and size using a pediatric acetate crown (Frasaco GmbH, Tettnang, Germany, No. 523, upper lateral incisor). Clinical and radiographic follow-up appointments were scheduled to monitor apical closure. After six months, the patient was asymptomatic, and the radiograph indicated progressive apical development (Figure 3a). Serial radiographs taken at one year (Figure 3b), two years (Figure 3c), three years (Figure 3d), four years (Figure 3e), five years (Figure 3f), and six years (Figure 3g) demonstrated continued asymptomatic status and apical repair, with evident thickening of the dentinal walls. A full CBCT scan confirmed incomplete apical closure of 2.2 mm in tooth 11 (Figure 3h).

### 2.3. Clinical Case 2

**Figure 4:**
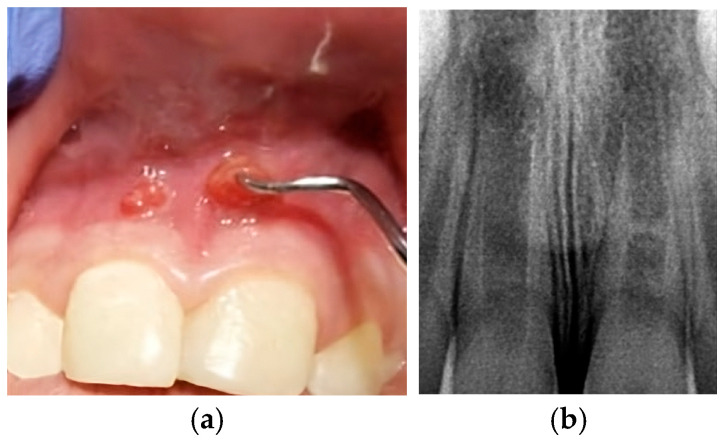
Clinical Case 2: (**a**) Intraoral photograph taken during the initial visit. (**b**) Periapical radiograph taken during the initial visit.

An 8-year-old girl presented for consultation after suffering trauma for more than one year of evolution. The mother reported that she had visited another dental clinic, where an apexification procedure had been attempted for one year on the maxillary right and left central incisors with Ca(OH)_2_ as intracanal medication, in addition to having undergone antibiotic therapy every three months during the last year. Clinical examination revealed fistulas in the vestibular area of teeth 11 and 21 (Figure 4a). Radiographic examination through periapical radiography showed open apices measuring 3.1 and 2.8, respectively (Nolla’s stage 8) [51], and thinning of the dentinal walls (Figure 4b). Based on the clinical and radiographic findings, a regenerative technique was planned for teeth 11 and 21, as detailed in Table 1.

**Figure 5:**
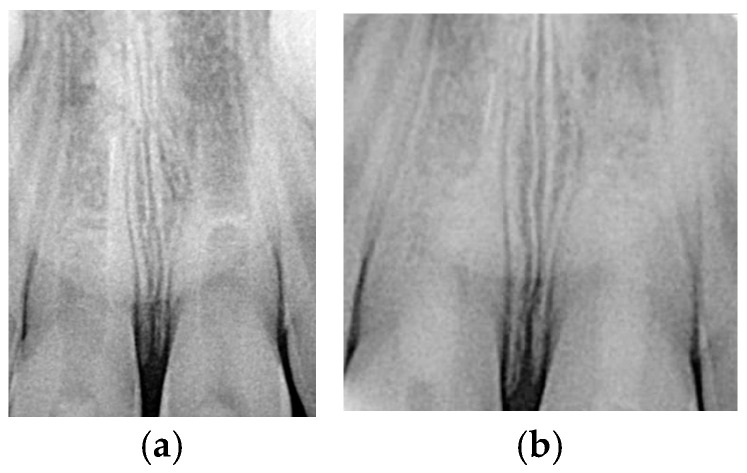
Clinical Case 2: (**a**) Access cavity was provisionally sealed with composite material at the first appointment. (**b**) Hermetic sealing with composite material at the second appointment.

At the first treatment appointment, an access cavity was prepared, and the canal was slowly irrigated with at least 20 mL of 2.5% NaOCl using a needle (Miraject-Endotec Luer 23G 0.6 × 25 m) positioned 1 mm from the apex. The canal was dried with paper points, and Ca(OH)_2_ was placed as intracanal medication using an appropriate instrument. The access cavity was provisionally sealed with composite material (Figure 5a).

At the second appointment, the access cavities were reopened, and a sterile file was used to go 2 mm beyond the working length to gently irritate the tissue and induce bleeding within the canal. Bleeding was allowed to stop at a level 3 mm below the cemento-enamel junction (CEJ) and was left for 15 min to allow clot formation. After 15 min, the presence of a blood clot was observed approximately 3 mm apical to the CEJ. Biodentine^®^ was carefully placed over the clot, followed by a cotton pellet and hermetic sealing with composite material (Figure 5b).

**Figure 6:**
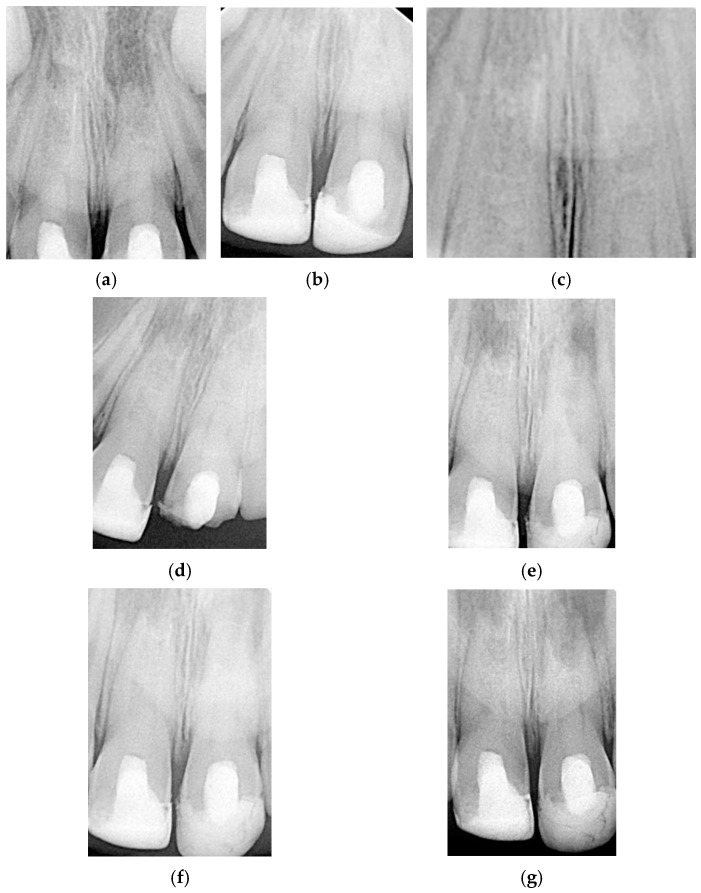
Clinical Case 2. Periapical radiograph taken at (**a**) 6-month follow-up, (**b**) 1-year follow-up, (**c**) 2-year follow-up, (**d**) 3-year follow-up, (**e**) 4-year follow-up, (**f**) 5-year follow-up, and (**g**) 6-year follow-up.

**Figure 7:**
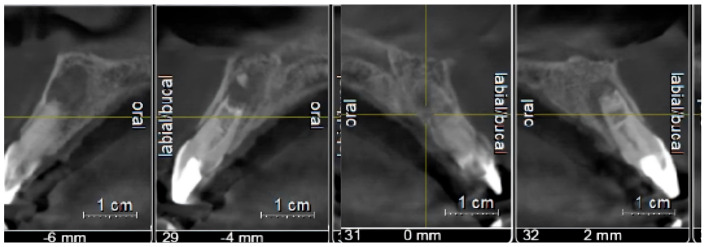
CBCT taken at 4-year follow-up. Axial section demonstrating the apical repair of teeth 11 and 21.

**Figure 8:**
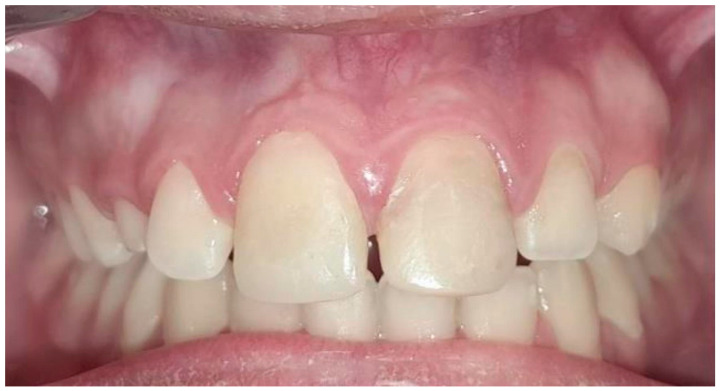
Intraoral photograph taken at the 6-year follow-up.

At the third appointment, an esthetic reconstruction of both teeth was performed. Clinical and radiographic follow-ups were scheduled to monitor apical repair. At the 6-month review, the patient was asymptomatic, and the radiograph showed continuous root apex development (Figure 6a). The sequence of radiographic follow-ups is shown at one year (Figure 6b), two years (Figure 6c), three years (Figure 6d), four years (Figure 6e), five years (Figure 6f), and six years (Figure 6g). The patient remained asymptomatic, with evidence of apical repair and thickening of the dentinal walls, as observed in the full CBCT performed at the 4-year follow-up. The scan showed an uncompleted apical closure of 2.3 mm in tooth 11 and 2 mm in tooth 21, as presented in Figure 7. Finally, Figure 8 illustrates how the sinus tract has resolved.

## 3. Discussion

The long-term success of the clinical cases presented underscores the need to preserve immature permanent teeth whenever feasible. This highlights the importance of prioritizing conservative, tooth-preserving strategies in young permanent teeth with incomplete root development. Early diagnosis and careful treatment planning, tailored to each patient’s individual needs, were crucial for achieving successful outcomes.

Case 1 involved a 7-year-old girl with a maxillary lateral incisor that became necrotic following an acute trauma. The tooth had an immature apex, measuring 3.7 mm in width, with thin dentinal walls. Case 2, by contrast, was an 8-year-old girl who presented with chronic infection in both maxillary central incisors over a year after trauma. Both incisors had been previously managed with a year-long Ca(OH)_2_ apexification attempt at another dental clinic, but radiographs still showed open apices (3.1 mm in tooth 11 and 2.8 mm in tooth 21) and persistent sinus tracts. These differing histories set the stage for somewhat different regenerative approaches: Case 1 was a primary regeneration treatment from the outset, whereas Case 2 represented a salvage of a failed apexification via regenerative therapy.

Both cases were ultimately managed in three visits, in line with the multi-appointment protocol recommended for REPs [6]. The first two visits were dedicated to disinfection and the regenerative procedure, and a third visit focused on final coronal restoration. In Case 1, the second appointment was scheduled after an interval of four weeks, whereas in Case 2, the first regenerative visit followed the protracted apexification attempt. AAE guidelines generally advise placing an intracanal medicament and delaying the second visit by about 1–4 weeks [6] to maximize disinfection, and both cases followed this principle. In Case 2, the initial Ca(OH)_2_ had been in place much longer, but once the decision for RET was made, the Ca(OH)_2_ was refreshed for two weeks before inducing bleeding. Evidently, both cases were treated under rubber dam isolation with minimal or no mechanical instrumentation of dentin, emphasizing chemical disinfection to avoid weakening the thin walls [52]. Each first visit concluded with a sterile temporary seal over the medicament, and each second visit employed regenerative techniques as described below.

In both cases, a blood clot scaffold technique, classic revascularization, was used to promote tissue ingrowth. After the canal was reopened during the second visit, the apical tissues were deliberately irritated with a sterile file passed just beyond the apex to induce bleeding into the canal. Bleeding was allowed to fill the canal up to 3 mm below the cementoenamel junction (CEJ) and then clotted for about 15 min. This induced blood clot serves as a natural scaffold rich in growth factors and stem cells from the apical papilla [52]. The choice of scaffold reflects current practice. Induced bleeding is the most common and recommended scaffold for REPs in immature teeth [52], given that more advanced scaffolds (platelet-rich plasma or platelet-rich fibrin) have so far shown mixed results without clear superiority [52]. Both cases achieved satisfactory canal bleeding, noting that the use of an anesthetic without a vasoconstrictor at the second visit can aid this, as per guidelines [52]. The creation of a stable intracanal blood clot was confirmed before sealing the root canal.

A key difference between the cases was the antimicrobial strategy during the first visit. Case 1 employed a triple antibiotic paste (TAP), prepared by mixing ciprofloxacin, metronidazole, and minocycline into a creamy paste (1:1:1 ratio), as originally described by Hoshino et al. [23]. This antibiotic mix was delivered into the canal with a lentulo spiral and left as an intracanal dressing. Case 2, on the other hand, utilized Ca(OH)_2_ as the sole intracanal medicament. Both approaches are supported by contemporary guidelines: the AAE permits either a low-concentration TAP or Ca(OH)_2_ for regenerative endodontics [6]. In Case 2, Ca(OH)_2_ was a natural choice since the patient’s teeth had already been medicated with it over the prior year; importantly, the failure of the initial apexification attempt was likely due to persistent infection rather than the ineffectiveness of Ca(OH)_2_ per se, since proper sealing and patient follow-up were lacking. Once the regenerative protocol was instituted, the infection was controlled, evidenced by the resolution of the sinus tracts.

After inducing the blood clot scaffold, a biomaterial was placed over the clot in the canal orifice to seal and protect it. In Case 1, a 3–4 mm layer of mineral trioxide aggregate (MTA) was gently packed just above the level of the clot (at 3 mm below CEJ), and the access was then double-sealed with a cotton pellet and bonded resin composite. In Case 2, Biodentine was used as the barrier material. Both materials achieve an airtight seal and provide a source of calcium ions that may stimulate hard tissue formation at the root apex. The choice of MTA in Case 1 versus Biodentine in Case 2 did not significantly alter the outcome, which aligns with studies finding both MTA and Biodentine to perform effectively as pulp space barriers in REPs, with no differences in healing or apical closure outcomes [53].

After placing the MTA or Biodentine barrier, both cases had the access cavities permanently restored with resin composite. At a third visit, the teeth received final aesthetic restorations (Case 1’s fractured lateral incisor was built up with a pediatric crown form, and Case 2’s incisors were restored to normal form). The coronal restorations ensured a hermetic seal to prevent reinfection, a critical factor for regenerative success [52].

Both cases demonstrated excellent clinical outcomes. By the first recall at 6 months, the patients were asymptomatic, with no pain, no tenderness to percussion, and resolution of sinus tracts, as seen in Figure 8. Throughout the subsequent follow-ups, both cases remained symptom-free with healthy soft tissues, satisfying the primary goal of regenerative therapy, elimination of symptoms and infection [6,52]. Notably, in Case 1, the more acute trauma case, there had been no pre-treatment sinus tract; in Case 2, the pre-treatment sinus tracts closed within a few months after REP, indicating successful infection control. Cold and electric pulp tests were periodically attempted, but no true vitality response was regained in any of the treated teeth, as is often the case in REPs. The lack of a positive pulp test does not equate failure; return of sensibility is a tertiary outcome and is not consistently achieved [52]. Studies report that only 50–60% of published REP cases show any positive pulp test, and even those might be responding via innervated vital tissues that are not actually new pulp tissue [52]. In both cases, the persistent negative pulp tests were expected, and the teeth still functioned normally and remained free of symptoms over the long term. In line with the current endodontic literature, the term “regenerative” is used clinically to describe treatment outcomes that promote periapical healing and continued root development; however, it should be acknowledged that such findings most likely represent repair processes, as true pulp–dentin regeneration can only be confirmed histologically [54].

CBCT imaging was selectively used when conventional radiographs were insufficient to assess three-dimensional root development and periapical healing. All scans were obtained using a limited field of view and pediatric protocols in accordance with ALARA/ALADAIP principles [55,56]. Radiographs and CBCT images revealed significant evidence of periapical healing and progressive apical closure in both cases. Periapical radiolucency resolution is observed within 6–12 months in successful regenerative cases [6], and our cases followed this pattern. By one year, both cases showed higher periapical bone density with no signs of lingering infection. From the 12-month to 24-month marks, continued changes in root dimensions became more apparent: the previously thin dentinal walls thickened appreciably, and the open apices began to close with hard tissue formation. This corresponds with the literature, which notes that increases in root wall width are often seen by 12–24 months post-treatment, even before obvious increases in root length [6]. Yearly radiographs from years 3 to 6 showed further incremental maturation. Importantly, CBCT at 4 years in Case 2 and at 6 years in Case 1 provided confirmation of apical repair and dentinal wall thickening in 3D, avoiding the superimposition issues of 2D radiographs [53].

Quantitatively, the differences in apical closure between the two cases were measured from the CBCT data at the final follow-up in Case 1 and at the 4-year follow-up in Case 2. In Case 1, the wide-open apex (initial diameter 3.7 mm) had narrowed to 2.2 mm by year 6, indicating roughly 1.5 mm of new hard tissue deposition forming an apical barrier. The root walls also thickened considerably along the canal length, improving the root’s fracture resistance. Although the apex was not fully closed to a normal (<1 mm) foramen, the formation of a smaller calcified bridge at the apex is a common and satisfactory outcome in regenerative cases [52]. The continued root development in Case 1 also slightly increased root length.

In Case 2, the two treated central incisors started with open apices of 3.1 mm (tooth 11) and 2.8 mm (tooth 21). After 4 years of healing, CBCT showed tooth 11’s apex had closed to about 2.3 mm, and the apex of tooth 21 had closed to about 2.0 mm. This corresponds to approximately 0.8 mm of apical closure in each tooth. The slightly lesser degree of narrowing compared to Case 1 might be related to the initial apical sizes, which were somewhat smaller to begin with, or simply individual biological variation. Importantly, both incisors in Case 2 also showed evident thickening of their dentinal walls over time, which is a critical sign of maturogenesis. The outcome in Case 2 can be deemed a success even without completely formed apices; what matters clinically is that a calcified barrier formed to seal the canal, the roots strengthened, and the periapical tissues healed. Both cases fortunately achieved substantial structural improvement, illustrating the potential of REP to induce significant root maturation beyond simple apex closure.

By the final 6-year follow-ups, both cases fulfilled the primary and secondary success criteria of regenerative endodontics: the teeth were asymptomatic with no signs of infection (primary goal), and radiographs/CBCT confirmed periapical healing along with increased root wall thickness and some length/apical closure (secondary goal) [52]. The tertiary goal (positive pulp vitality) was not met, but as discussed, this is an ideal that is not essential for a clinically successful outcome [52]. No adverse events, such as external root resorption or crown discoloration, were observed in either case over the 6-year period. In Case 1, despite the use of minocycline-containing TAP and MTA, the crown of the lateral incisor remained acceptably natural in color, likely aided by sealing the access and perhaps the short duration of TAP contact, but this case underscores why modern protocols suggest minimizing minocycline use to avoid potential staining [6,52]. Both cases also avoided any root fractures, a testament to the fact that the roots not only survived but were biomechanically reinforced by new hard tissue, addressing a major concern with long-term Ca(OH)_2_-treated teeth, which can become fragile [52]

After regenerative treatment in immature permanent teeth, several patterns of apical closure can be observed, depending on the initial condition of the tooth, as well as the technique and materials used. Apical closure may be complete [57,58,59] or partial [58,59,60]; a calcified apical barrier can develop [58,61,62]; continued root development is sometimes observed [57,59,62,63]; or, less frequently, marked obliteration or calcification of the canal is seen [58,60]. Teeth with less initial root development have a lower probability of achieving complete apical closure, although this outcome is still possible after regenerative treatment [49]. The type of apical closure observed in both clinical cases was the formation of a calcified apical barrier.

These two cases highlight the scientific and clinical relevance of regenerative endodontic therapy as a viable treatment for necrotic immature teeth while also illustrating how different clinical approaches can lead to similarly successful outcomes.

## 4. Conclusions

These cases highlight the importance of a case-by-case decision-making process, in which the selection of materials and therapeutic strategies is tailored to the individual clinical scenario. The long-term favorable outcomes observed over a follow-up period exceeding six years support the potential durability of biologically based regenerative endodontic procedures in necrotic immature teeth. Although limited by the descriptive nature of case-based evidence, these findings are consistent with current endodontic guidelines and previously published evidence, which recognize regenerative approaches as a viable treatment option aimed at promoting periapical healing and continued root development.

## Figures and Tables

**Table 1:** Comparison of procedures performed and radiographic findings in both clinical cases.

	TEETH	Irrigation Protocol	Intracanal Medication	Biomaterial	Apex Measurement (mm) Before Treatment	Apex Measurement (mm) After Treatment	Type of Apical Closure
Case 1	12	17% EDTA NaOCl	TAP	MTA	3.7 mm	2.2 mm	Calcified apical barrier
Case 2	11/21	17% EDTA NaOCl	Ca(OH)_2_	BIODENTINE^®^	3.1 mm/2.8 mm	2.3 mm/2 mm	Calcified apical barrier

TAP: triple antibiotic paste; EDTA: ethylenediaminetetraacetic acid; NaOCl: sodium hypochlorite; Ca(OH)_2_: calcium hydroxide; MTA: mineral trioxide aggregate; Biodentine: Septodont, Saint-Maur-des-Fiossés, France.

## Data Availability

The original contributions presented in this study are included in the article. Further inquiries can be directed to the corresponding author.

## Abbreviations

The following abbreviations are used in this manuscript:

CEJ	Cemento-enamel junction
Ca(OH)_2_	Calcium hydroxide
CBCT	Cone beam computed tomography
RET	Regenerative endodontic treatment
AAE	American Association of Endodontists
ESE	European Society of Endodontology
REPs	Regenerative endodontic procedures
EDTA	Ethylenediaminetetraacetic acid
TAP	Triple antibiotic paste
NaOCl	Sodium hypochlorite
